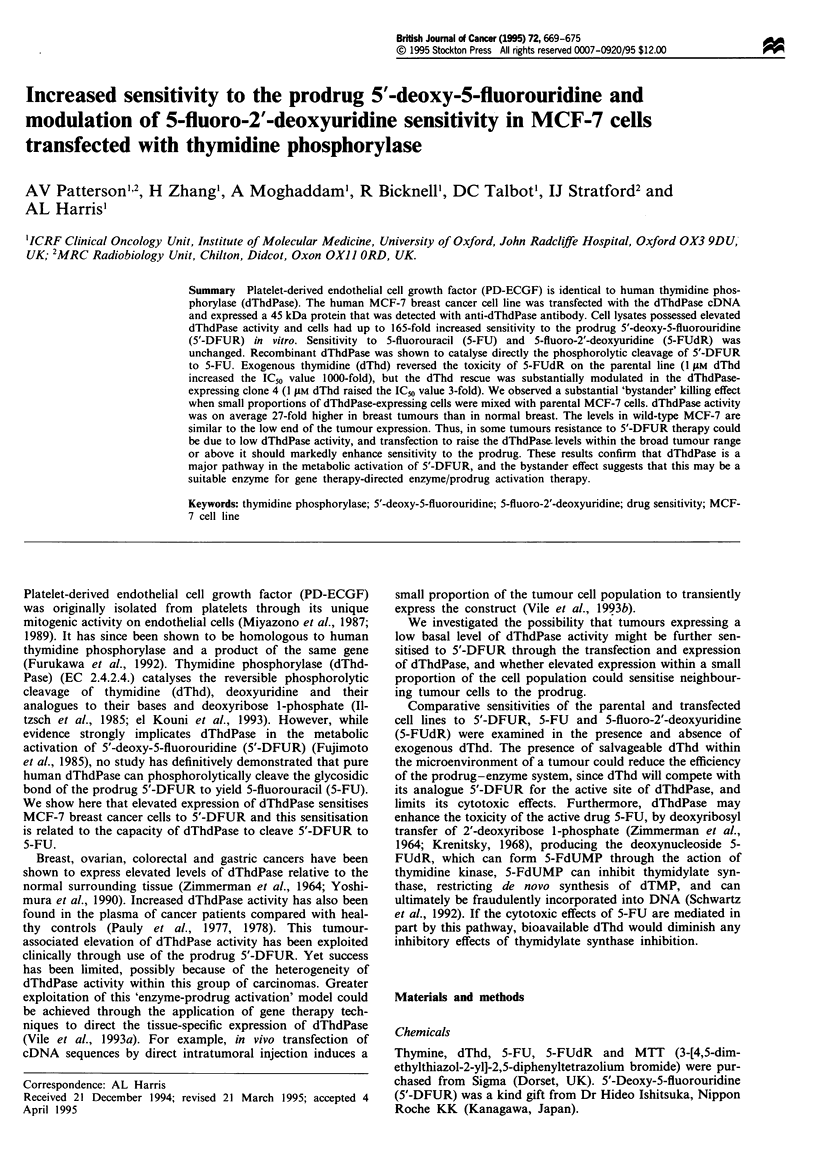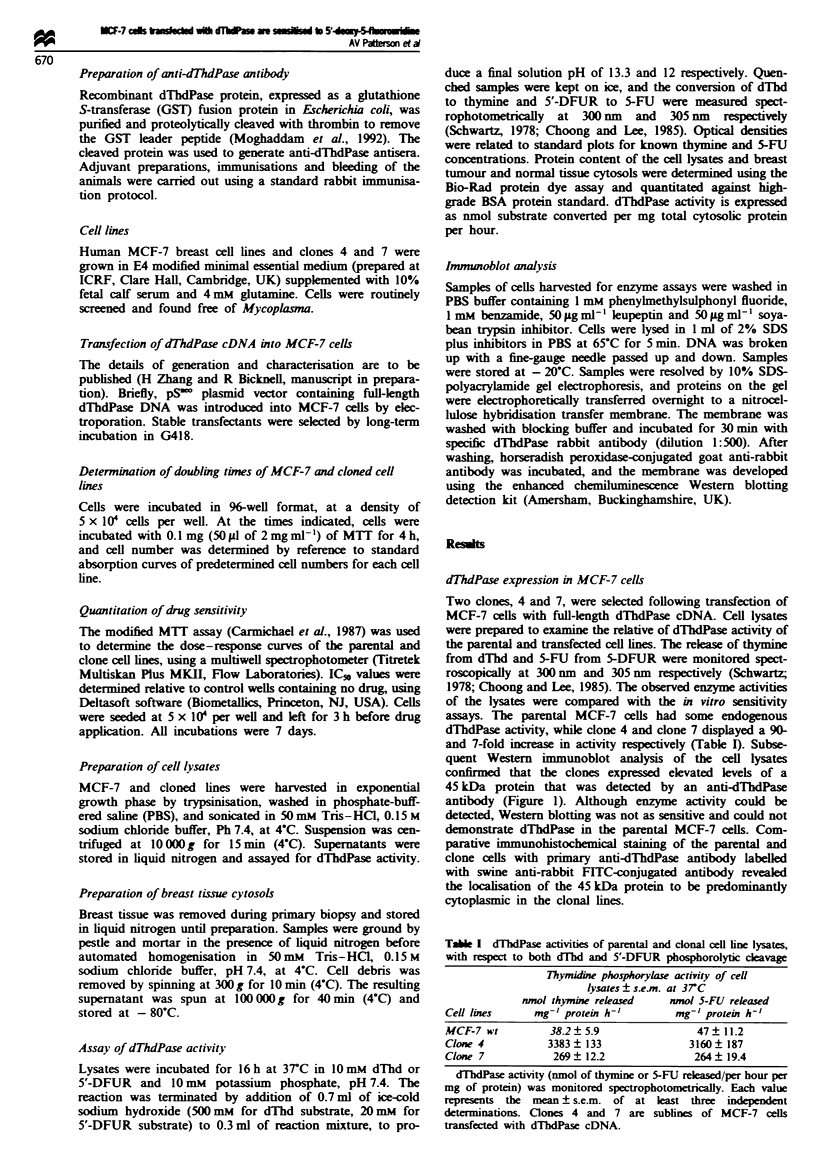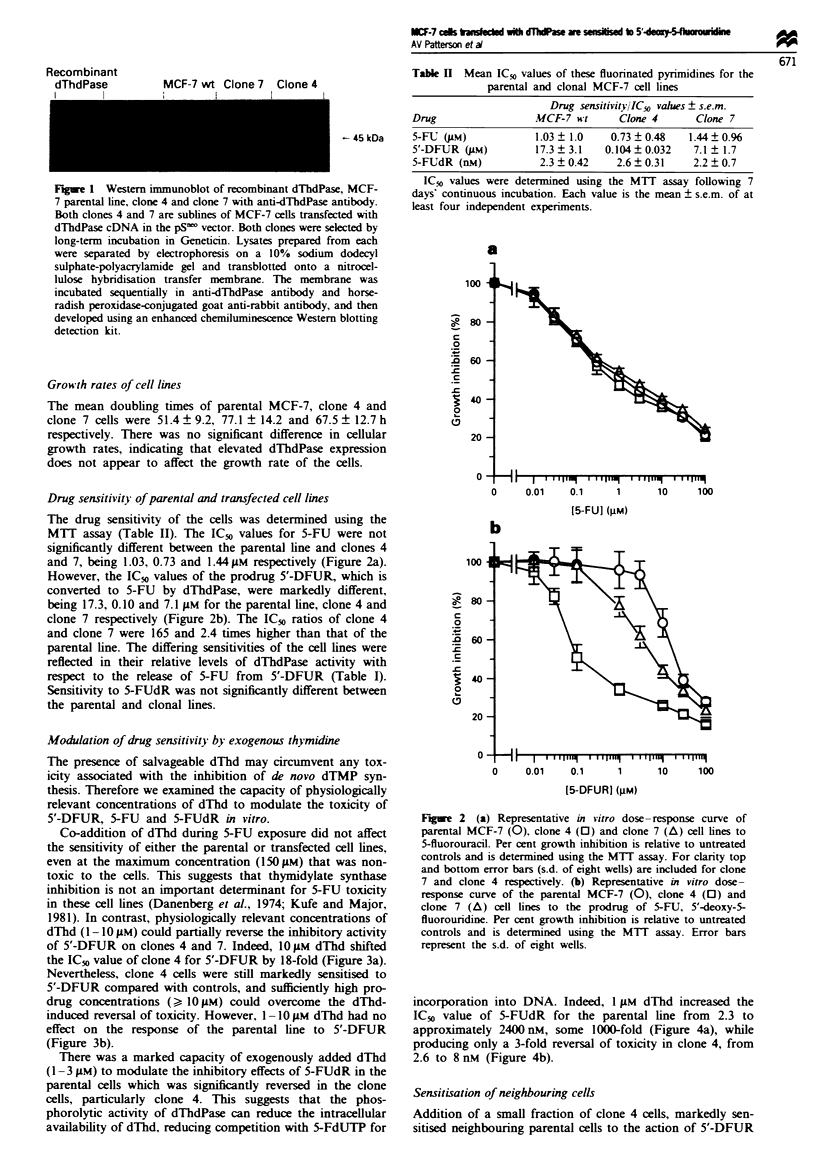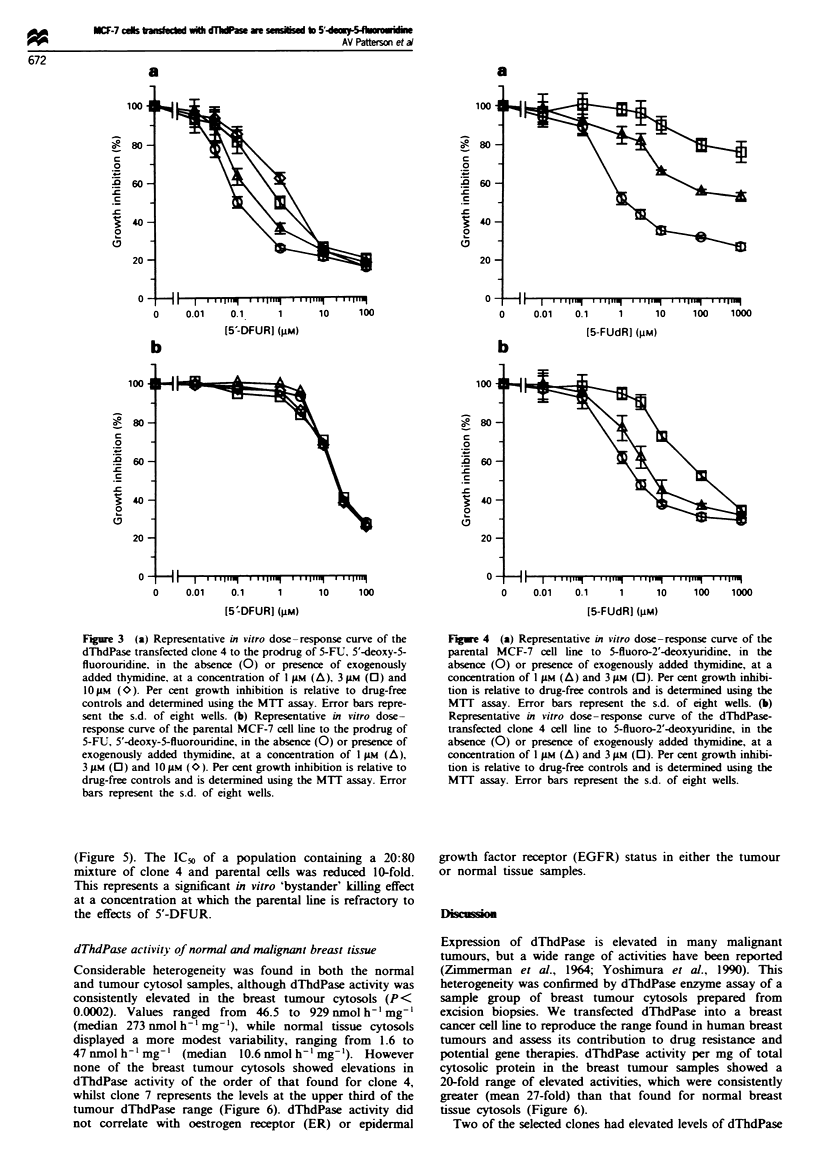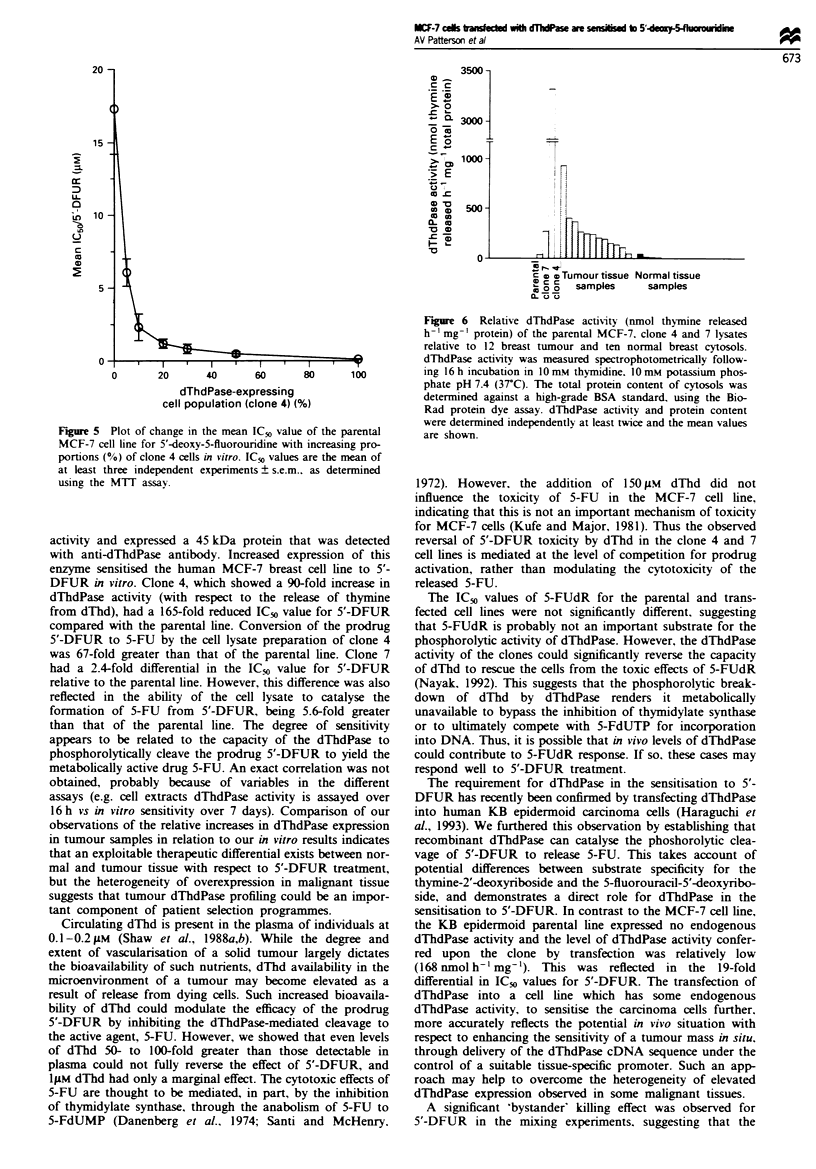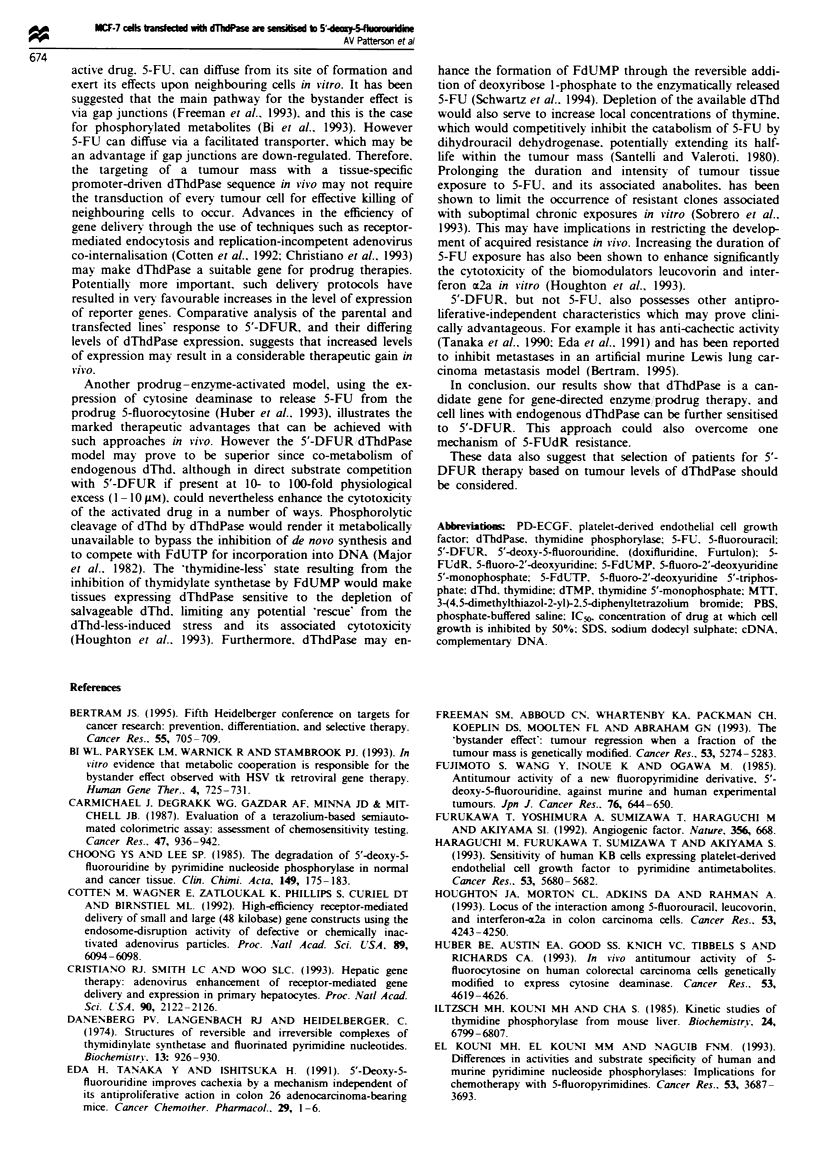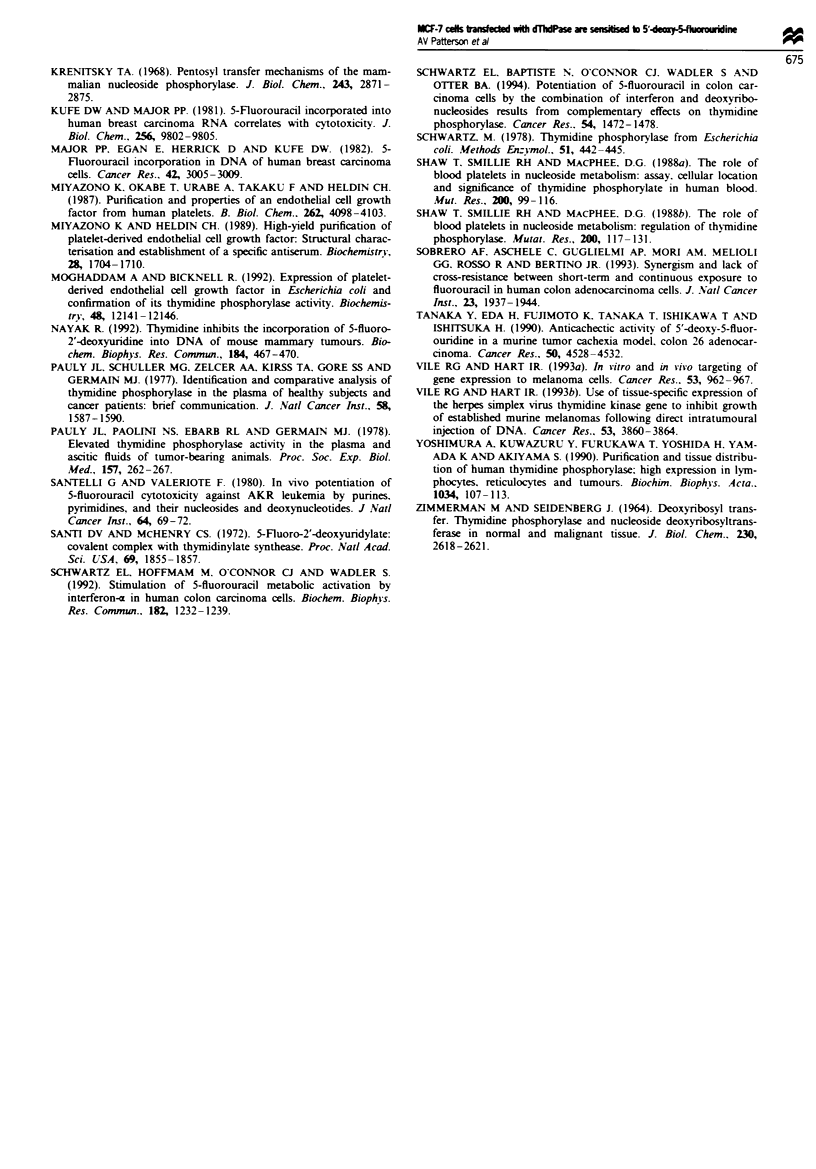# Increased sensitivity to the prodrug 5'-deoxy-5-fluorouridine and modulation of 5-fluoro-2'-deoxyuridine sensitivity in MCF-7 cells transfected with thymidine phosphorylase.

**DOI:** 10.1038/bjc.1995.392

**Published:** 1995-09

**Authors:** A. V. Patterson, H. Zhang, A. Moghaddam, R. Bicknell, D. C. Talbot, I. J. Stratford, A. L. Harris

**Affiliations:** ICRF Clinical Oncology Unit, University of Oxford, John Radcliffe Hospital, UK.

## Abstract

**Images:**


British Journal of Cancer (1995) 72, 669-675
© 1995 Stockton Press All rights reserved 0007-0920/95 $12.00

### Increased sensitivity to the prodrug 5′-deoxy-5-fluorouridine and modulation of 5-fluoro-2′-deoxyuridine sensitivity in MCF-7 cells transfected with thymidine phosphorylase

AV Patterson[1,2], H Zhang[1], A Moghaddam[1], R Bicknell[1], DC Talbot[1], IJ Stratford[2] and AL Harris[1]

[1]*ICRF Clinical Oncology Unit, Institute of Molecular Medicine, University of Oxford, John Radcliffe Hospital, Oxford OX3 9DU, UK;* [2]*MRC Radiobiology Unit, Chilton, Didcot, Oxon OX11 0RD, UK.*

**Summary** Platelet-derived endothelial cell growth factor (PD-ECGF) is identical to human thymidine phosphorylase (dThdPase). The human MCF-7 breast cancer cell line was transfected with the dThdPase cDNA and expressed a 45 kDa protein that was detected with anti-dThdPase antibody. Cell lysates possessed elevated dThdPase activity and cells had up to 165-fold increased sensitivity to the prodrug 5′-deoxy-5-fluorouridine (5′-DFUR) *in vitro*. Sensitivity to 5-fluorouracil (5-FU) and 5-fluoro-2′-deoxyuridine (5-FUdR) was unchanged. Recombinant dThdPase was shown to catalyse directly the phosphorolytic cleavage of 5′-DFUR to 5-FU. Exogenous thymidine (dThd) reversed the toxicity of 5-FUdR on the parental line (1 μM dThd increased the $IC_{50}$ value 1000-fold), but the dThd rescue was substantially modulated in the dThdPase-expressing clone 4 (1 μM dThd raised the $IC_{50}$ value 3-fold). We observed a substantial 'bystander' killing effect when small proportions of dThdPase-expressing cells were mixed with parental MCF-7 cells. dThdPase activity was on average 27-fold higher in breast tumours than in normal breast. The levels in wild-type MCF-7 are similar to the low end of the tumour expression. Thus, in some tumours resistance to 5′-DFUR therapy could be due to low dThdPase activity, and transfection to raise the dThdPase levels within the broad tumour range or above it should markedly enhance sensitivity to the prodrug. These results confirm that dThdPase is a major pathway in the metabolic activation of 5′-DFUR, and the bystander effect suggests that this may be a suitable enzyme for gene therapy-directed enzyme/prodrug activation therapy.

**Keywords:** thymidine phosphorylase; 5′-deoxy-5-fluorouridine; 5-fluoro-2′-deoxyuridine; drug sensitivity; MCF-7 cell line

Platelet-derived endothelial cell growth factor (PD-ECGF) was originally isolated from platelets through its unique mitogenic activity on endothelial cells (Miyazono *et al.*, 1987; 1989). It has since been shown to be homologous to human thymidine phosphorylase and a product of the same gene (Furukawa *et al.*, 1992). Thymidine phosphorylase (dThdPase) (EC 2.4.2.4.) catalyses the reversible phosphorolytic cleavage of thymidine (dThd), deoxyuridine and their analogues to their bases and deoxyribose 1-phosphate (Iltzsch *et al.*, 1985; el Kouni *et al.*, 1993). However, while evidence strongly implicates dThdPase in the metabolic activation of 5′-deoxy-5-fluorouridine (5′-DFUR) (Fujimoto *et al.*, 1985), no study has definitively demonstrated that pure human dThdPase can phosphorolytically cleave the glycosidic bond of the prodrug 5′-DFUR to yield 5-fluorouracil (5-FU). We show here that elevated expression of dThdPase sensitises MCF-7 breast cancer cells to 5′-DFUR and this sensitisation is related to the capacity of dThdPase to cleave 5′-DFUR to 5-FU.

Breast, ovarian, colorectal and gastric cancers have been shown to express elevated levels of dThdPase relative to the normal surrounding tissue (Zimmerman *et al.*, 1964; Yoshimura *et al.*, 1990). Increased dThdPase activity has also been found in the plasma of cancer patients compared with healthy controls (Pauly *et al.*, 1977, 1978). This tumour-associated elevation of dThdPase activity has been exploited clinically through use of the prodrug 5′-DFUR. Yet success has been limited, possibly because of the heterogeneity of dThdPase activity within this group of carcinomas. Greater exploitation of this 'enzyme-prodrug activation' model could be achieved through the application of gene therapy techniques to direct the tissue-specific expression of dThdPase (Vile *et al.*, 1993*a*). For example, *in vivo* transfection of cDNA sequences by direct intratumoral injection induces a small proportion of the tumour cell population to transiently express the construct (Vile *et al.*, 1993*b*).

We investigated the possibility that tumours expressing a low basal level of dThdPase activity might be further sensitised to 5′-DFUR through the transfection and expression of dThdPase, and whether elevated expression within a small proportion of the cell population could sensitise neighbouring tumour cells to the prodrug.

Comparative sensitivities of the parental and transfected cell lines to 5′-DFUR, 5-FU and 5-fluoro-2′-deoxyuridine (5-FUdR) were examined in the presence and absence of exogenous dThd. The presence of salvageable dThd within the microenvironment of a tumour could reduce the efficiency of the prodrug–enzyme system, since dThd will compete with its analogue 5′-DFUR for the active site of dThdPase, and limits its cytotoxic effects. Furthermore, dThdPase may enhance the toxicity of the active drug 5-FU, by deoxyribosyl transfer of 2′-deoxyribose 1-phosphate (Zimmerman *et al.*, 1964; Krenitsky, 1968), producing the deoxynucleoside 5-FUdR, which can form 5-FdUMP through the action of thymidine kinase, 5-FdUMP can inhibit thymidylate synthase, restricting *de novo* synthesis of dTMP, and can ultimately be fraudulently incorporated into DNA (Schwartz *et al.*, 1992). If the cytotoxic effects of 5-FU are mediated in part by this pathway, bioavailable dThd would diminish any inhibitory effects of thymidylate synthase inhibition.

### Materials and methods

### *Chemicals*

Thymine, dThd, 5-FU, 5-FUdR and MTT (3-[4,5-dimethylthiazol-2-yl]-2,5-diphenyltetrazolium bromide) were purchased from Sigma (Dorset, UK). 5′-Deoxy-5-fluorouridine (5′-DFUR) was a kind gift from Dr Hideo Ishitsuka, Nippon Roche KK (Kanagawa, Japan).

Correspondence: AL Harris
Received 21 December 1994; revised 21 March 1995; accepted 4 April 1995

*Preparation of anti-dThdPase antibody*

Recombinant dThdPase protein, expressed as a glutathione S-transferase (GST) fusion protein in *Escherichia coli*, was purified and proteolytically cleaved with thrombin to remove the GST leader peptide (Moghaddam *et al.*, 1992). The cleaved protein was used to generate anti-dThdPase antisera. Adjuvant preparations, immunisations and bleeding of the animals were carried out using a standard rabbit immunisation protocol.

*Cell lines*

Human MCF-7 breast cell lines and clones 4 and 7 were grown in E4 modified minimal essential medium (prepared at ICRF, Clare Hall, Cambridge, UK) supplemented with 10% fetal calf serum and 4 mM glutamine. Cells were routinely screened and found free of *Mycoplasma*.

*Transfection of dThdPase cDNA into MCF-7 cells*

The details of generation and characterisation are to be published (H Zhang and R Bicknell, manuscript in preparation). Briefly, $pS^{neo}$ plasmid vector containing full-length dThdPase DNA was introduced into MCF-7 cells by electroporation. Stable transfectants were selected by long-term incubation in G418.

*Determination of doubling times of MCF-7 and cloned cell lines*

Cells were incubated in 96-well format, at a density of $5 \times 10^4$ cells per well. At the times indicated, cells were incubated with 0.1 mg (50 µl of 2 mg ml$^{-1}$) of MTT for 4 h, and cell number was determined by reference to standard absorption curves of predetermined cell numbers for each cell line.

*Quantitation of drug sensitivity*

The modified MTT assay (Carmichael *et al.*, 1987) was used to determine the dose–response curves of the parental and clone cell lines, using a multiwell spectrophotometer (Titretek Multiskan Plus MKII, Flow Laboratories). $IC_{50}$ values were determined relative to control wells containing no drug, using Deltasoft software (Biometallics, Princeton, NJ, USA). Cells were seeded at $5 \times 10^4$ per well and left for 3 h before drug application. All incubations were 7 days.

*Preparation of cell lysates*

MCF-7 and cloned lines were harvested in exponential growth phase by trypsinisation, washed in phosphate-buffered saline (PBS), and sonicated in 50 mM Tris–HCl, 0.15 M sodium chloride buffer, Ph 7.4, at 4°C. Suspension was centrifuged at 10 000 *g* for 15 min (4°C). Supernatants were stored in liquid nitrogen and assayed for dThdPase activity.

*Preparation of breast tissue cytosols*

Breast tissue was removed during primary biopsy and stored in liquid nitrogen until preparation. Samples were ground by pestle and mortar in the presence of liquid nitrogen before automated homogenisation in 50 mM Tris–HCl, 0.15 M sodium chloride buffer, pH 7.4, at 4°C. Cell debris was removed by spinning at 300 *g* for 10 min (4°C). The resulting supernatant was spun at 100 000 *g* for 40 min (4°C) and stored at − 80°C.

*Assay of dThdPase activity*

Lysates were incubated for 16 h at 37°C in 10 mM dThd or 5'-DFUR and 10 mM potassium phosphate, pH 7.4. The reaction was terminated by addition of 0.7 ml of ice-cold sodium hydroxide (500 mM for dThd substrate, 20 mM for 5'-DFUR substrate) to 0.3 ml of reaction mixture, to produce a final solution pH of 13.3 and 12 respectively. Quenched samples were kept on ice, and the conversion of dThd to thymine and 5'-DFUR to 5-FU were measured spectrophotometrically at 300 nm and 305 nm respectively (Schwartz, 1978; Choong and Lee, 1985). Optical densities were related to standard plots for known thymine and 5-FU concentrations. Protein content of the cell lysates and breast tumour and normal tissue cytosols were determined using the Bio-Rad protein dye assay and quantitated against high-grade BSA protein standard. dThdPase activity is expressed as nmol substrate converted per mg total cytosolic protein per hour.

*Immunoblot analysis*

Samples of cells harvested for enzyme assays were washed in PBS buffer containing 1 mM phenylmethylsulphonyl fluoride, 1 mM benzamide, 50 µg ml$^{-1}$ leupeptin and 50 µg ml$^{-1}$ soyabean trypsin inhibitor. Cells were lysed in 1 ml of 2% SDS plus inhibitors in PBS at 65°C for 5 min. DNA was broken up with a fine-gauge needle passed up and down. Samples were stored at − 20°C. Samples were resolved by 10% SDS-polyacrylamide gel electrophoresis, and proteins on the gel were electrophoretically transferred overnight to a nitrocellulose hybridisation transfer membrane. The membrane was washed with blocking buffer and incubated for 30 min with specific dThdPase rabbit antibody (dilution 1:500). After washing, horseradish peroxidase-conjugated goat anti-rabbit antibody was incubated, and the membrane was developed using the enhanced chemiluminescence Western blotting detection kit (Amersham, Buckinghamshire, UK).

### Results

*dThdPase expression in MCF-7 cells*

Two clones, 4 and 7, were selected following transfection of MCF-7 cells with full-length dThdPase cDNA. Cell lysates were prepared to examine the relative of dThdPase activity of the parental and transfected cell lines. The release of thymine from dThd and 5-FU from 5-DFUR were monitored spectroscopically at 300 nm and 305 nm respectively (Schwartz; 1978; Choong and Lee, 1985). The observed enzyme activities of the lysates were compared with the *in vitro* sensitivity assays. The parental MCF-7 cells had some endogenous dThdPase activity, while clone 4 and clone 7 displayed a 90- and 7-fold increase in activity respectively (Table I). Subsequent Western immunoblot analysis of the cell lysates confirmed that the clones expressed elevated levels of a 45 kDa protein that was detected by an anti-dThdPase antibody (Figure 1). Although enzyme activity could be detected, Western blotting was not as sensitive and could not demonstrate dThdPase in the parental MCF-7 cells. Comparative immunohistochemical staining of the parental and clone cells with primary anti-dThdPase antibody labelled with swine anti-rabbit FITC-conjugated antibody revealed the localisation of the 45 kDa protein to be predominantly cytoplasmic in the clonal lines.

**Table I** dThdPase activities of parental and clonal cell line lysates, with respect to both dThd and 5'-DFUR phosphorolytic cleavage

| Cell lines | *Thymidine phosphorylase activity of cell lysates ± s.e.m. at 37°C* nmol thymine released mg$^{-1}$ protein h$^{-1}$ | nmol 5-FU released mg$^{-1}$ protein h$^{-1}$ |
|---|---|---|
| *MCF-7 wt* | 38.2 ± 5.9 | 47 ± 11.2 |
| *Clone 4* | 3383 ± 133 | 3160 ± 187 |
| *Clone 7* | 269 ± 12.2 | 264 ± 19.4 |

dThdPase activity (nmol of thymine or 5-FU released/per hour per mg of protein) was monitored spectrophotometrically. Each value represents the mean ± s.e.m. of at least three independent determinations. Clones 4 and 7 are sublines of MCF-7 cells transfected with dThdPase cDNA.

**Figure 1** Western immunoblot of recombinant dThdPase, MCF-7 parental line, clone 4 and clone 7 with anti-dThdPase antibody. Both clones 4 and 7 are sublines of MCF-7 cells transfected with dThdPase cDNA in the pS$^{neo}$ vector. Both clones were selected by long-term incubation in Geneticin. Lysates prepared from each were separated by electrophoresis on a 10% sodium dodecyl sulphate-polyacrylamide gel and transblotted onto a nitrocellulose hybridisation transfer membrane. The membrane was incubated sequentially in anti-dThdPase antibody and horseradish peroxidase-conjugated goat anti-rabbit antibody, and then developed using an enhanced chemiluminescence Western blotting detection kit.

*Growth rates of cell lines*

The mean doubling times of parental MCF-7, clone 4 and clone 7 cells were 51.4 ± 9.2, 77.1 ± 14.2 and 67.5 ± 12.7 h respectively. There was no significant difference in cellular growth rates, indicating that elevated dThdPase expression does not appear to affect the growth rate of the cells.

*Drug sensitivity of parental and transfected cell lines*

The drug sensitivity of the cells was determined using the MTT assay (Table II). The $IC_{50}$ values for 5-FU were not significantly different between the parental line and clones 4 and 7, being 1.03, 0.73 and 1.44 μM respectively (Figure 2a). However, the $IC_{50}$ values of the prodrug 5′-DFUR, which is converted to 5-FU by dThdPase, were markedly different, being 17.3, 0.10 and 7.1 μM for the parental line, clone 4 and clone 7 respectively (Figure 2b). The $IC_{50}$ ratios of clone 4 and clone 7 were 165 and 2.4 times higher than that of the parental line. The differing sensitivities of the cell lines were reflected in their relative levels of dThdPase activity with respect to the release of 5-FU from 5′-DFUR (Table I). Sensitivity to 5-FUdR was not significantly different between the parental and clonal lines.

*Modulation of drug sensitivity by exogenous thymidine*

The presence of salvageable dThd may circumvent any toxicity associated with the inhibition of *de novo* dTMP synthesis. Therefore we examined the capacity of physiologically relevant concentrations of dThd to modulate the toxicity of 5′-DFUR, 5-FU and 5-FUdR *in vitro*.

Co-addition of dThd during 5-FU exposure did not affect the sensitivity of either the parental or transfected cell lines, even at the maximum concentration (150 μM) that was non-toxic to the cells. This suggests that thymidylate synthase inhibition is not an important determinant for 5-FU toxicity in these cell lines (Danenberg *et al.*, 1974; Kufe and Major, 1981). In contrast, physiologically relevant concentrations of dThd (1–10 μM) could partially reverse the inhibitory activity of 5′-DFUR on clones 4 and 7. Indeed, 10 μM dThd shifted the $IC_{50}$ value of clone 4 for 5′-DFUR by 18-fold (Figure 3a). Nevertheless, clone 4 cells were still markedly sensitised to 5′-DFUR compared with controls, and sufficiently high prodrug concentrations (⩾ 10 μM) could overcome the dThd-induced reversal of toxicity. However, 1–10 μM dThd had no effect on the response of the parental line to 5′-DFUR (Figure 3b).

There was a marked capacity of exogenously added dThd (1–3 μM) to modulate the inhibitory effects of 5-FUdR in the parental cells which was significantly reversed in the clone cells, particularly clone 4. This suggests that the phosphorolytic activity of dThdPase can reduce the intracellular availability of dThd, reducing competition with 5-FdUTP for

**Table II** Mean $IC_{50}$ values of these fluorinated pyrimidines for the parental and clonal MCF-7 cell lines

| | Drug sensitivity/$IC_{50}$ values ± s.e.m. | | |
|---|---|---|---|
| *Drug* | *MCF-7 wt* | *Clone 4* | *Clone 7* |
| 5-FU (μM) | 1.03 ± 1.0 | 0.73 ± 0.48 | 1.44 ± 0.96 |
| 5′-DFUR (μM) | 17.3 ± 3.1 | 0.104 ± 0.032 | 7.1 ± 1.7 |
| 5-FUdR (nM) | 2.3 ± 0.42 | 2.6 ± 0.31 | 2.2 ± 0.7 |

$IC_{50}$ values were determined using the MTT assay following 7 days' continuous incubation. Each value is the mean ± s.e.m. of at least four independent experiments.

**Figure 2** (**a**) Representative *in vitro* dose–response curve of parental MCF-7 (○), clone 4 (□) and clone 7 (△) cell lines to 5-fluorouracil. Per cent growth inhibition is relative to untreated controls and is determined using the MTT assay. For clarity top and bottom error bars (s.d. of eight wells) are included for clone 7 and clone 4 respectively. (**b**) Representative *in vitro* dose–response curve of the parental MCF-7 (○), clone 4 (□) and clone 7 (△) cell lines to the prodrug of 5-FU, 5′-deoxy-5-fluorouridine. Per cent growth inhibition is relative to untreated controls and is determined using the MTT assay. Error bars represent the s.d. of eight wells.

incorporation into DNA. Indeed, 1 μM dThd increased the $IC_{50}$ value of 5-FUdR for the parental line from 2.3 to approximately 2400 nM, some 1000-fold (Figure 4a), while producing only a 3-fold reversal of toxicity in clone 4, from 2.6 to 8 nM (Figure 4b).

*Sensitisation of neighbouring cells*

Addition of a small fraction of clone 4 cells, markedly sensitised neighbouring parental cells to the action of 5′-DFUR

**Figure 3** (**a**) Representative *in vitro* dose–response curve of the dThdPase transfected clone 4 to the prodrug of 5-FU, 5'-deoxy-5-fluorouridine, in the absence (○) or presence of exogenously added thymidine, at a concentration of 1 μM (△), 3 μM (□) and 10 μM (◇). Per cent growth inhibition is relative to drug-free controls and determined using the MTT assay. Error bars represent the s.d. of eight wells. (**b**) Representative *in vitro* dose–response curve of the parental MCF-7 cell line to the prodrug of 5-FU, 5'-deoxy-5-fluorouridine, in the absence (○) or presence of exogenously added thymidine, at a concentration of 1 μM (△), 3 μM (□) and 10 μM (◇). Per cent growth inhibition is relative to drug-free controls and is determined using the MTT assay. Error bars represent the s.d. of eight wells.

**Figure 4** (**a**) Representative *in vitro* dose–response curve of the parental MCF-7 cell line to 5-fluoro-2'-deoxyuridine, in the absence (○) or presence of exogenously added thymidine, at a concentration of 1 μM (△) and 3 μM (□). Per cent growth inhibition is relative to drug-free controls and is determined using the MTT assay. Error bars represent the s.d. of eight wells. (**b**) Representative *in vitro* dose–response curve of the dThdPase-transfected clone 4 cell line to 5-fluoro-2'-deoxyuridine, in the absence (○) or presence of exogenously added thymidine, at a concentration of 1 μM (△) and 3 μM (□). Per cent growth inhibition is relative to drug-free controls and is determined using the MTT assay. Error bars represent the s.d. of eight wells.

(Figure 5). The $IC_{50}$ of a population containing a 20:80 mixture of clone 4 and parental cells was reduced 10-fold. This represents a significant *in vitro* 'bystander' killing effect at a concentration at which the parental line is refractory to the effects of 5'-DFUR.

### *dThdPase activity of normal and malignant breast tissue*

Considerable heterogeneity was found in both the normal and tumour cytosol samples, although dThdPase activity was consistently elevated in the breast tumour cytosols ($P <$ 0.0002). Values ranged from 46.5 to 929 nmol $h^{-1}$ $mg^{-1}$ (median 273 nmol $h^{-1}$ $mg^{-1}$), while normal tissue cytosols displayed a more modest variability, ranging from 1.6 to 47 nmol $h^{-1}$ $mg^{-1}$ (median 10.6 nmol $h^{-1}$ $mg^{-1}$). However none of the breast tumour cytosols showed elevations in dThdPase activity of the order of that found for clone 4, whilst clone 7 represents the levels at the upper third of the tumour dThdPase range (Figure 6). dThdPase activity did not correlate with oestrogen receptor (ER) or epidermal growth factor receptor (EGFR) status in either the tumour or normal tissue samples.

### Discussion

Expression of dThdPase is elevated in many malignant tumours, but a wide range of activities have been reported (Zimmerman *et al.*, 1964; Yoshimura *et al.*, 1990). This heterogeneity was confirmed by dThdPase enzyme assay of a sample group of breast tumour cytosols prepared from excision biopsies. We transfected dThdPase into a breast cancer cell line to reproduce the range found in human breast tumours and assess its contribution to drug resistance and potential gene therapies. dThdPase activity per mg of total cytosolic protein in the breast tumour samples showed a 20-fold range of elevated activities, which were consistently greater (mean 27-fold) than that found for normal breast tissue cytosols (Figure 6).

Two of the selected clones had elevated levels of dThdPase

**Figure 5** Plot of change in the mean $IC_{50}$ value of the parental MCF-7 cell line for 5'-deoxy-5-fluorouridine with increasing proportions (%) of clone 4 cells *in vitro*. $IC_{50}$ values are the mean of at least three independent experiments ± s.e.m., as determined using the MTT assay.

**Figure 6** Relative dThdPase activity (nmol thymine released $h^{-1}$ $mg^{-1}$ protein) of the parental MCF-7, clone 4 and 7 lysates relative to 12 breast tumour and ten normal breast cytosols. dThdPase activity was measured spectrophotometrically following 16 h incubation in 10 mM thymidine, 10 mM potassium phosphate pH 7.4 (37°C). The total protein content of cytosols was determined against a high-grade BSA standard, using the Bio-Rad protein dye assay. dThdPase activity and protein content were determined independently at least twice and the mean values are shown.

activity and expressed a 45 kDa protein that was detected with anti-dThdPase antibody. Increased expression of this enzyme sensitised the human MCF-7 breast cell line to 5'-DFUR *in vitro*. Clone 4, which showed a 90-fold increase in dThdPase activity (with respect to the release of thymine from dThd), had a 165-fold reduced $IC_{50}$ value for 5'-DFUR compared with the parental line. Conversion of the prodrug 5'-DFUR to 5-FU by the cell lysate preparation of clone 4 was 67-fold greater than that of the parental line. Clone 7 had a 2.4-fold differential in the $IC_{50}$ value for 5'-DFUR relative to the parental line. However, this difference was also reflected in the ability of the cell lysate to catalyse the formation of 5-FU from 5'-DFUR, being 5.6-fold greater than that of the parental line. The degree of sensitivity appears to be related to the capacity of the dThdPase to phosphorolytically cleave the prodrug 5'-DFUR to yield the metabolically active drug 5-FU. An exact correlation was not obtained, probably because of variables in the different assays (e.g. cell extracts dThdPase activity is assayed over 16 h *vs in vitro* sensitivity over 7 days). Comparison of our observations of the relative increases in dThdPase expression in tumour samples in relation to our *in vitro* results indicates that an exploitable therapeutic differential exists between normal and tumour tissue with respect to 5'-DFUR treatment, but the heterogeneity of overexpression in malignant tissue suggests that tumour dThdPase profiling could be an important component of patient selection programmes.

Circulating dThd is present in the plasma of individuals at 0.1–0.2 μM (Shaw *et al*., 1988*a,b*). While the degree and extent of vascularisation of a solid tumour largely dictates the bioavailability of such nutrients, dThd availability in the microenvironment of a tumour may become elevated as a result of release from dying cells. Such increased bioavailability of dThd could modulate the efficacy of the prodrug 5'-DFUR by inhibiting the dThdPase-mediated cleavage to the active agent, 5-FU. However, we showed that even levels of dThd 50- to 100-fold greater than those detectable in plasma could not fully reverse the effect of 5'-DFUR, and 1μM dThd had only a marginal effect. The cytotoxic effects of 5-FU are thought to be mediated, in part, by the inhibition of thymidylate synthase, through the anabolism of 5-FU to 5-FdUMP (Danenberg *et al*., 1974; Santi and McHenry, 1972). However, the addition of 150 μM dThd did not influence the toxicity of 5-FU in the MCF-7 cell line, indicating that this is not an important mechanism of toxicity for MCF-7 cells (Kufe and Major, 1981). Thus the observed reversal of 5'-DFUR toxicity by dThd in the clone 4 and 7 cell lines is mediated at the level of competition for prodrug activation, rather than modulating the cytotoxicity of the released 5-FU.

The $IC_{50}$ values of 5-FUdR for the parental and transfected cell lines were not significantly different, suggesting that 5-FUdR is probably not an important substrate for the phosphorolytic activity of dThdPase. However, the dThdPase activity of the clones could significantly reverse the capacity of dThd to rescue the cells from the toxic effects of 5-FUdR (Nayak, 1992). This suggests that the phosphorolytic breakdown of dThd by dThdPase renders it metabolically unavailable to bypass the inhibition of thymidylate synthase or to ultimately compete with 5-FdUTP for incorporation into DNA. Thus, it is possible that *in vivo* levels of dThdPase could contribute to 5-FUdR response. If so, these cases may respond well to 5'-DFUR treatment.

The requirement for dThdPase in the sensitisation to 5'-DFUR has recently been confirmed by transfecting dThdPase into human KB epidermoid carcinoma cells (Haraguchi *et al*., 1993). We furthered this observation by establishing that recombinant dThdPase can catalyse the phoshorolytic cleavage of 5'-DFUR to release 5-FU. This takes account of potential differences between substrate specificity for the thymine-2'-deoxyriboside and the 5-fluorouracil-5'-deoxyriboside, and demonstrates a direct role for dThdPase in the sensitisation to 5'-DFUR. In contrast to the MCF-7 cell line, the KB epidermoid parental line expressed no endogenous dThdPase activity and the level of dThdPase activity conferred upon the clone by transfection was relatively low (168 nmol $h^{-1}$ $mg^{-1}$). This was reflected in the 19-fold differential in $IC_{50}$ values for 5'-DFUR. The transfection of dThdPase into a cell line which has some endogenous dThdPase activity, to sensitise the carcinoma cells further, more accurately reflects the potential *in vivo* situation with respect to enhancing the sensitivity of a tumour mass *in situ*, through delivery of the dThdPase cDNA sequence under the control of a suitable tissue-specific promoter. Such an approach may help to overcome the heterogeneity of elevated dThdPase expression observed in some malignant tissues.

A significant 'bystander' killing effect was observed for 5'-DFUR in the mixing experiments, suggesting that the active drug, 5-FU, can diffuse from its site of formation and exert its effects upon neighbouring cells *in vitro*. It has been suggested that the main pathway for the bystander effect is via gap junctions (Freeman *et al*., 1993), and this is the case for phosphorylated metabolites (Bi *et al*., 1993). However 5-FU can diffuse via a facilitated transporter, which may be an advantage if gap junctions are down-regulated. Therefore, the targeting of a tumour mass with a tissue-specific promoter-driven dThdPase sequence *in vivo* may not require the transduction of every tumour cell for effective killing of neighbouring cells to occur. Advances in the efficiency of gene delivery through the use of techniques such as receptor-mediated endocytosis and replication-incompetent adenovirus co-internalisation (Cotten *et al*., 1992; Christiano *et al*., 1993) may make dThdPase a suitable gene for prodrug therapies. Potentially more important, such delivery protocols have resulted in very favourable increases in the level of expression of reporter genes. Comparative analysis of the parental and transfected lines' response to 5'-DFUR, and their differing levels of dThdPase expression, suggests that increased levels of expression may result in a considerable therapeutic gain *in vivo*.

Another prodrug–enzyme-activated model, using the expression of cytosine deaminase to release 5-FU from the prodrug 5-fluorocytosine (Huber *et al*., 1993), illustrates the marked therapeutic advantages that can be achieved with such approaches *in vivo*. However the 5'-DFUR/dThdPase model may prove to be superior since co-metabolism of endogenous dThd, although in direct substrate competition with 5'-DFUR if present at 10- to 100-fold physiological excess (1–10 μM), could nevertheless enhance the cytotoxicity of the activated drug in a number of ways. Phosphorolytic cleavage of dThd by dThdPase would render it metabolically unavailable to bypass the inhibition of *de novo* synthesis and to compete with FdUTP for incorporation into DNA (Major *et al*., 1982). The 'thymidine-less' state resulting from the inhibition of thymidylate synthetase by FdUMP would make tissues expressing dThdPase sensitive to the depletion of salvageable dThd, limiting any potential 'rescue' from the dThd-less-induced stress and its associated cytotoxicity (Houghton *et al*., 1993). Furthermore, dThdPase may enhance the formation of FdUMP through the reversible addition of deoxyribose 1-phosphate to the enzymatically released 5-FU (Schwartz *et al*., 1994). Depletion of the available dThd would also serve to increase local concentrations of thymine, which would competitively inhibit the catabolism of 5-FU by dihydrouracil dehydrogenase, potentially extending its half-life within the tumour mass (Santelli and Valeroti, 1980). Prolonging the duration and intensity of tumour tissue exposure to 5-FU, and its associated anabolites, has been shown to limit the occurrence of resistant clones associated with suboptimal chronic exposures *in vitro* (Sobrero *et al*., 1993). This may have implications in restricting the development of acquired resistance *in vivo*. Increasing the duration of 5-FU exposure has also been shown to enhance significantly the cytotoxicity of the biomodulators leucovorin and interferon α2a *in vitro* (Houghton *et al*., 1993).

5'-DFUR, but not 5-FU, also possesses other antiproliferative-independent characteristics which may prove clinically advantageous. For example it has anti-cachectic activity (Tanaka *et al*., 1990; Eda *et al*., 1991) and has been reported to inhibit metastases in an artificial murine Lewis lung carcinoma metastasis model (Bertram, 1995).

In conclusion, our results show that dThdPase is a candidate gene for gene-directed enzyme/prodrug therapy, and cell lines with endogenous dThdPase can be further sensitised to 5'-DFUR. This approach could also overcome one mechanism of 5-FUdR resistance.

These data also suggest that selection of patients for 5'-DFUR therapy based on tumour levels of dThdPase should be considered.

**Abbreviations:** PD-ECGF, platelet-derived endothelial cell growth factor; dThdPase, thymidine phosphorylase; 5-FU, 5-fluorouracil; 5'-DFUR, 5'-deoxy-5-fluorouridine, (doxifluridine, Furtulon); 5-FUdR, 5-fluoro-2'-deoxyuridine; 5-FdUMP, 5-fluoro-2'-deoxyuridine 5'-monophosphate; 5-FdUTP, 5-fluoro-2'-deoxyuridine 5'-triphosphate; dThd, thymidine; dTMP, thymidine 5'-monophosphate; MTT, 3-(4,5-dimethylthiazol-2-yl)-2,5-diphenyltetrazolium bromide; PBS, phosphate-buffered saline; $IC_{50}$, concentration of drug at which cell growth is inhibited by 50%; SDS, sodium dodecyl sulphate; cDNA, complementary DNA.

### References

BERTRAM JS. (1995). Fifth Heidelberger conference on targets for cancer research: prevention, differentiation, and selective therapy. *Cancer Res*., **55**, 705–709.

BI WL, PARYSEK LM, WARNICK R AND STAMBROOK PJ. (1993). *In vitro* evidence that metabolic cooperation is responsible for the bystander effect observed with HSV tk retroviral gene therapy. *Human Gene Ther*., **4**, 725–731.

CARMICHAEL J, DEGRAKK WG, GAZDAR AF, MINNA JD & MITCHELL JB. (1987). Evaluation of a terazolium-based semiautomated colorimetric assay: assessment of chemosensitivity testing. *Cancer Res*., **47**, 936–942.

CHOONG YS AND LEE SP. (1985). The degradation of 5'-deoxy-5-fluorouridine by pyrimidine nucleoside phosphorylase in normal and cancer tissue. *Clin. Chimi. Acta*, **149**, 175–183.

COTTEN M, WAGNER E, ZATLOUKAL K, PHILLIPS S, CURIEL DT AND BIRNSTIEL ML. (1992). High-efficiency receptor-mediated delivery of small and large (48 kilobase) gene constructs using the endosome-disruption activity of defective or chemically inactivated adenovirus particles. *Proc. Natl Acad. Sci. USA*, **89**, 6094–6098.

CRISTIANO RJ, SMITH LC AND WOO SLC. (1993). Hepatic gene therapy: adenovirus enhancement of receptor-mediated gene delivery and expression in primary hepatocytes. *Proc. Natl Acad. Sci. USA*, **90**, 2122–2126.

DANENBERG PV, LANGENBACH RJ AND HEIDELBERGER, C. (1974). Structures of reversible and irreversible complexes of thymidinylate synthetase and fluorinated pyrimidine nucleotides. *Biochemistry*, **13**: 926–930.

EDA H, TANAKA Y AND ISHITSUKA H. (1991). 5'-Deoxy-5-fluorouridine improves cachexia by a mechanism independent of its antiproliferative action in colon 26 adenocarcinoma-bearing mice. *Cancer Chemother. Pharmacol*., **29**, 1–6.

FREEMAN SM, ABBOUD CN, WHARTENBY KA, PACKMAN CH, KOEPLIN DS, MOOLTEN FL AND ABRAHAM GN (1993). The 'bystander effect': tumour regression when a fraction of the tumour mass is genetically modified. *Cancer Res*., **53**, 5274–5283.

FUJIMOTO S, WANG Y, INOUE K AND OGAWA M. (1985). Antitumour activity of a new fluoropyrimidine derivative, 5'-deoxy-5-fluorouridine, against murine and human experimental tumours. *Jpn J. Cancer Res*., **76**, 644–650.

FURUKAWA T, YOSHIMURA A, SUMIZAWA T, HARAGUCHI M AND AKIYAMA SI. (1992). Angiogenic factor. *Nature*, **356**, 668.

HARAGUCHI M, FURUKAWA T, SUMIZAWA T AND AKIYAMA S. (1993). Sensitivity of human KB cells expressing platelet-derived endothelial cell growth factor to pyrimidine antimetabolites. *Cancer Res*., **53**, 5680–5682.

HOUGHTON JA, MORTON CL, ADKINS DA AND RAHMAN A. (1993). Locus of the interaction among 5-fluorouracil, leucovorin, and interferon-α2a in colon carcinoma cells. *Cancer Res*., **53**, 4243–4250.

HUBER BE, AUSTIN EA, GOOD SS, KNICH VC, TIBBELS S AND RICHARDS CA. (1993). *In vivo* antitumour activity of 5-fluorocytosine on human colorectal carcinoma cells genetically modified to express cytosine deaminase. *Cancer Res*., **53**, 4619–4626.

ILTZSCH MH, KOUNI MH AND CHA S. (1985). Kinetic studies of thymidine phosphorylase from mouse liver. *Biochemistry*, **24**, 6799–6807.

EL KOUNI MH, EL KOUNI MM AND NAGUIB FNM. (1993). Differences in activities and substrate specificity of human and murine pyridimine nucleoside phosphorylases: Implications for chemotherapy with 5-fluoropyrimidines. *Cancer Res*., **53**, 3687–3693.

KRENITSKY TA. (1968). Pentosyl transfer mechanisms of the mammalian nucleoside phosphorylase. *J. Biol. Chem.*, **243**, 2871-2875.

KUFE DW AND MAJOR PP. (1981). 5-Fluorouracil incorporated into human breast carcinoma RNA correlates with cytotoxicity. *J. Biol. Chem.*, **256**, 9802-9805.

MAJOR PP. EGAN E. HERRICK D AND KUFE DW. (1982). 5-Fluorouracil incorporation in DNA of human breast carcinoma cells. *Cancer Res.*, **42**, 3005-3009.

MIYAZONO K. OKABE T. URABE A. TAKAKU F AND HELDIN CH. (1987). Purification and properties of an endothelial cell growth factor from human platelets. *B. Biol. Chem.*, **262**, 4098-4103.

MIYAZONO K AND HELDIN CH. (1989). High-yield purification of platelet-derived endothelial cell growth factor: Structural characterisation and establishment of a specific antiserum. *Biochemistry*, **28**, 1704-1710.

MOGHADDAM A AND BICKNELL R. (1992). Expression of platelet-derived endothelial cell growth factor in *Escherichia coli* and confirmation of its thymidine phosphorylase activity. *Biochemistry*, **48**, 12141-12146.

NAYAK R. (1992). Thymidine inhibits the incorporation of 5-fluoro-2'-deoxyuridine into DNA of mouse mammary tumours. *Biochem. Biophys. Res. Commun.*, **184**, 467-470.

PAULY JL. SCHULLER MG. ZELCER AA. KIRSS TA. GORE SS AND GERMAIN MJ. (1977). Identification and comparative analysis of thymidine phosphorylase in the plasma of healthy subjects and cancer patients: brief communication. *J. Natl Cancer Inst.*, **58**, 1587-1590.

PAULY JL. PAOLINI NS. EBARB RL AND GERMAIN MJ. (1978). Elevated thymidine phosphorylase activity in the plasma and ascitic fluids of tumor-bearing animals. *Proc. Soc. Exp. Biol. Med.*, **157**, 262-267.

SANTELLI G AND VALERIOTE F. (1980). In vivo potentiation of 5-fluorouracil cytotoxicity against AKR leukemia by purines, pyrimidines, and their nucleosides and deoxynucleotides. *J Natl Cancer Inst.*, **64**, 69-72.

SANTI DV AND MCHENRY CS. (1972). 5-Fluoro-2'-deoxyuridylate: covalent complex with thymidinylate synthease. *Proc. Natl Acad. Sci. USA*, **69**, 1855-1857.

SCHWARTZ EL. HOFFMAM M. O'CONNOR CJ AND WADLER S. (1992). Stimulation of 5-fluorouracil metabolic activation by interferon-α in human colon carcinoma cells. *Biochem. Biophys. Res. Commun.*, **182**, 1232-1239.

SCHWARTZ EL. BAPTISTE N. O'CONNOR CJ. WADLER S AND OTTER BA. (1994). Potentiation of 5-fluorouracil in colon carcinoma cells by the combination of interferon and deoxyribonucleosides results from complementary effects on thymidine phosphorylase. *Cancer Res.*, **54**, 1472-1478.

SCHWARTZ. M. (1978). Thymidine phosphorylase from *Escherichia coli*. *Methods Enzymol.*, **51**, 442-445.

SHAW T. SMILLIE RH AND MACPHEE. D.G. (1988*a*). The role of blood platelets in nucleoside metabolism: assay, cellular location and significance of thymidine phosphorylate in human blood. *Mut. Res.*, **200**, 99-116.

SHAW T. SMILLIE RH AND MACPHEE. D.G. (1988*b*). The role of blood platelets in nucleoside metabolism: regulation of thymidine phosphorylase. *Mutat. Res.*, **200**, 117-131.

SOBRERO AF. ASCHELE C. GUGLIELMI AP. MORI AM. MELIOLI GG. ROSSO R AND BERTINO JR. (1993). Synergism and lack of cross-resistance between short-term and continuous exposure to fluorouracil in human colon adenocarcinoma cells. *J. Natl Cancer Inst.*, **23**, 1937-1944.

TANAKA Y. EDA H. FUJIMOTO K. TANAKA T. ISHIKAWA T AND ISHITSUKA H. (1990). Anticachectic activity of 5'-deoxy-5-fluorouridine in a murine tumor cachexia model, colon 26 adenocarcinoma. *Cancer Res.*, **50**, 4528-4532.

VILE RG AND HART IR. (1993*a*). *In vitro* and *in vivo* targeting of gene expression to melanoma cells. *Cancer Res.*, **53**, 962-967.

VILE RG AND HART IR. (1993*b*). Use of tissue-specific expression of the herpes simplex virus thymidine kinase gene to inhibit growth of established murine melanomas following direct intratumoural injection of DNA. *Cancer Res.*, **53**, 3860-3864.

YOSHIMURA A. KUWAZURU Y. FURUKAWA T. YOSHIDA H. YAMADA K AND AKIYAMA S. (1990). Purification and tissue distribution of human thymidine phosphorylase; high expression in lymphocytes, reticulocytes and tumours. *Biochim. Biophys. Acta.*, **1034**, 107-113.

ZIMMERMAN M AND SEIDENBERG J. (1964). Deoxyribosyl transfer. Thymidine phosphorylase and nucleoside deoxyribosyltransferase in normal and malignant tissue. *J. Biol. Chem.*, **230**, 2618-2621.